# Persistence and progression of staphylococcal infection in the presence of public goods

**DOI:** 10.1038/s41522-020-00168-2

**Published:** 2020-11-27

**Authors:** Urvish Trivedi, Cody Fell, Jonas S. Madsen, Jake Everett, Mette Burmølle, Kendra P. Rumbaugh, Søren J. Sørensen

**Affiliations:** 1 Section of Microbiology, Department of Biology, Faculty of Science, University of Copenhagen, 2100 Copenhagen, Denmark; 2 Department of Surgery, Texas Tech University Health Sciences Center, Lubbock, TX 79430 USA

**Keywords:** Pathogens, Microbial communities

## Abstract

*Staphylococcus aureus* is a prominent etiological agent of suppurative abscesses. In principle, abscess formation and purulent exudate are classical physiological features of healing and tissue repair. However, *S. aureus* deploys two coagulases that can usurp this classical host response and form distinct abscess lesions. Here, we establish that during coinfection with coagulase producers and non-producers, coagulases are shared public goods that contribute to staphylococcal persistence, abscess formation, and disease progression. Coagulase-negative mutants that do not produce the public goods themselves are able to exploit those cooperatively secreted by producers and thereby thrive during coinfection at the expense of others. This study shows the importance of social interactions among pathogens concerning clinical outcomes.

*Staphylococcus aureus* engages a large set of its adhesin/invasins, pore-forming toxins, superantigens, and immune evasion factors upon entering tissues or the bloodstream via trauma, surgical wounds, or medical devices^1,2^. These virulence factors contribute to *S. aureus* colonization, dissemination, and tissue damage^3^. Using an in vitro infection model and ex vivo human blood, we recently showed that *S. aureus* coagulases, staphylocoagulase (Coa) and von Willebrand factor binding protein (vWbp), act as “public goods” during bloodstream-related infection^4^. These two hemostasis factors usurp the otherwise physiological coagulation cascade by independently activating the central coagulation zymogen, prothrombin (ProT), in a non-proteolytic manner; the resulting staphylothrombin complexes (ProT•Coa and ProT•vWbp) then convert soluble fibrinogen to the polymerized insoluble fibrin strands and generate clots^5–8^. By doing so, the resulting fibrin layers shield staphylococci from antimicrobials and host immune factors^4^. Interestingly, we found that this protective effect was conferred onto the entire staphylococcal community, including cheats that do not cooperatively produce coagulases but still benefit from those made by others. The significance of our findings was made relevant to bloodstream-related infections. However, during bacteremia, *S. aureus* can also disseminate to blood filtration organs such as the kidneys and accumulate in the arcuate renal arteries, causing infarcts and abscesses^9–11^.

In principle, the events of abscess formation are part of a default host response initiated upon chemical trauma, physical insult, or the entry of foreign biological substance into tissues^12,13^. Any one of these instances triggers the release of proinflammatory cytokines and extravasation of polymorphonuclear leukocytes and macrophages to the site of injury or infection. The area of inflammation is delineated from healthy tissue by fibrin deposition, accompanied by phagocytic degradation of damaged tissue; the liquefactive necrosis is eventually drained to organ surfaces to promote healing^14–16^. However, with its ability to co-opt the host-mediated pathway of fibrin deposition, *S. aureus* is now recognized as the most frequent cause of skin and soft tissue infections^2^. Earlier work highlighted the stages of staphylococcal abscess formation and the role that sortase A-anchored surface proteins and coagulases play^17,18^. However, phenotypes of low expression levels, loss-of-function mutations, and/or complete deficiency of coagulases are observed among clinical isolates^19–25^. Here, we dissect the relative contribution of coagulases from a microbial interactions perspective—whether or not they can function as public goods in mixed *S. aureus* communities of coagulase-positive and -negative isogenic strains during systemic spread and abscess formation. Using an in vivo murine model, we investigate whether access to coagulases, either through cooperation or cheating, influences staphylococcal persistence during infection and its clinical significance. Our model organisms include a community-acquired methicillin-resistant *S. aureus*, USA300 LAC that produces coagulases; a Δ*coa* mutant that does not produce Coa; and a Δ*coa*Δ*vwbp* double mutant that does not produce Coa and vWbp. The Δ*coa* and Δ*coa*Δ*vwbp* mutants represent cheats that do not produce the public goods of interest, whereas LAC represents producers.

A sub-lethal suspension of *S. aureus* was injected into the retro-orbital sinus. Monoinfection cohorts were infected with LAC, Δ*coa*, or Δ*coa*Δ*vwbp*. Whereas coinfection cohorts were infected with a 1:1 mixture of LAC and Δ*coa*, or LAC and Δ*coa*Δ*vwbp*. The route of blood flow from the retro-orbital sinus^26^ ensures that high blood levels of the injectate are achieved rapidly^27^, but the majority of inoculum disappears from the vasculature within 6 hours^17^. In addition to passive transport via blood flow, the remaining *staphylococci* can survive within leukocytes and use them as a vehicle to escape the vasculature and taxi into uninfected regions of host tissues^28^.

We gross examined the kidneys and spleens on Day 15 post infection to assess the contribution of coagulases towards the hematogenous spread of *S. aureus* and the establishment of metastatic infections. The kidneys of the mice monoinfected with producers displayed signs of pyelonephritis and tissue ischemia (Fig. 1a and Supplementary Fig. S1a); similar pathology was observed for those coinfected with both producers and cheats. The majority of these mice had visible abscesses on either one or both of their kidneys (Fig. 1b and Supplementary Fig. S1a). In contrast, six out of nine mice in both groups infected with only cheats had normal kidneys with no ischemic tissue or visible abscesses (Fig. 1b and Supplementary Fig. S1a). Thus, abscessed kidneys were not simply a result of the default host response to invading pathogens, indicating that the action of coagulases was required for staphylococcal persistence and abscess lesions sustained.

**Fig. 1:**
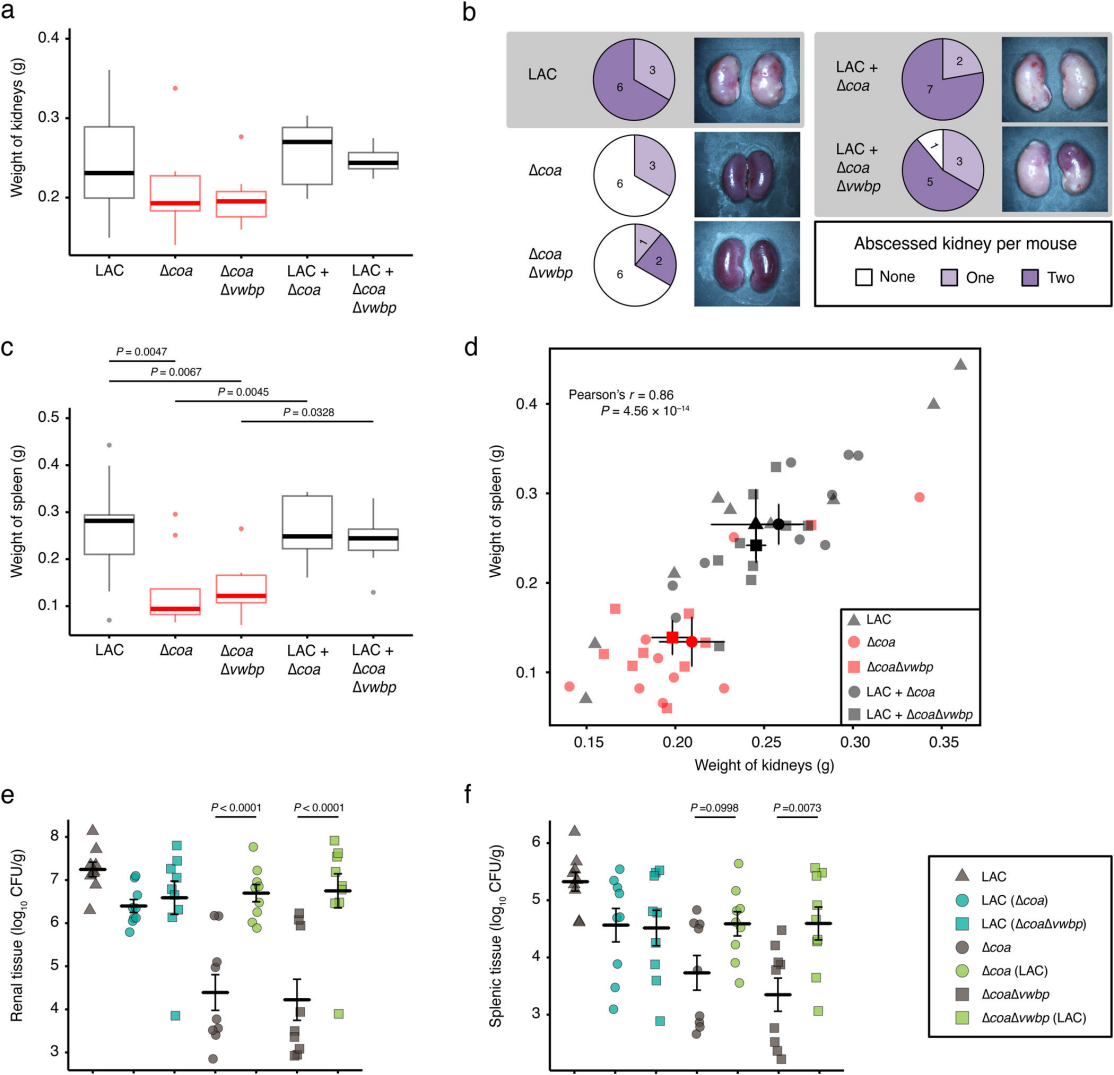
Coagulases contribute to staphylococcal persistence, disease progression, and organ inflammation. Mice (*n* = 9 for each cohort) were injected in their retro-orbital sinus with 1 × 10^6^ CFU of LAC, Δ*coa*, or Δ*coa*Δ*vwbp*; and cocultures of LAC and Δ*coa*, or LAC and Δ*coa*Δ*vwbp*. On Day 15 post infection, the kidneys and spleens were excised, gross-examined, photographed, homogenized and spread on selective agar for enumeration of CFUs. The data represent two trials. **a** Pyelonephritis plotted as the weight of each pair of kidneys (adjusted *P* values calculated using general linear hypotheses test with manual contrast). **b** Number of mice that had abscesses on either one or both of their kidneys; images are of kidneys representing the pathology observed within each cohort. **c** Splenomegaly plotted as the weight of each spleen (adjusted *P* values calculated using general linear hypotheses test with manual contrast). **d** Plot showing Pearson correlation between splenomegaly and pyelonephritis. Staphylococcal load in **e** kidneys and **f** spleens plotted as log_10_ CFU per gram of tissue (adjusted *P* values calculated using general linear hypotheses test with manual contrast, error bars denote ±SEM). Boxplot elements are: center line–median; box limits–upper and lower quartiles; whiskers–1.5×interquartile range; points–outliers.

The spleens of the mice infected with only producers, and coinfected with producers and cheats displayed signs of splenomegaly (Fig. 1c and Supplementary Fig. S1b). Whereas those infected with only cheats had normal spleens without inflammation. No splenic abscesses were observed for any of the cohorts (Supplementary Fig. S1b). However, there was a strong correlation (Pearson’s *r* = 0.86 *P* = 4.56 × 10^–14^) of corresponding pathology between the two organs, where the mice that had abscessed and inflamed kidneys also had an inflamed spleen (Fig. 1d). An explanation for this is that in addition to being an immunological filter, the spleen is a reservoir and hub of the mononuclear phagocyte system; therefore, immune hyperplasia in the spleen is a result of staphylococcal persistence in the kidneys. Furthermore, staphylococci captured within a fibrin meshwork, may also disseminate as septic emboli and resist opsonophagocytic clearance by host immune cells. Taken together, the host’s inability to clear the infection owing to the action of coagulases results in exaggerated inflammation and disease progression.

We next assessed the bacterial load in both organs to see whether coagulases secreted by producers could rescue cheats from phagocytosis and allow them to proliferate in host tissues. Mice that were monoinfected with LAC had a higher bacterial load in their kidneys and spleen compared with those monoinfected with Δ*coa* or Δ*coa*Δ*vwbp* (*P* < 0.001) (Fig. 1e, f), indicating that cheats were impaired in their ability to persist and replicate in host tissue owing to the absence of coagulases. In contrast, there was a dramatic improvement in Δ*coa* and Δ*coa*Δ*vwbp* survival when they were coinfected together with LAC (Fig. 1e, f). This trend was observed for both organs. In addition, there was a slight decrease in the LAC bacterial load in the kidneys and spleen during coinfection compared to monoinfection. These data indicate that the cooperative secretion of coagulases can enhance staphylococcal fitness in host tissues. However, cheats can exploit the public goods secreted by producers without incurring the metabolic costs associated with producing coagulases and thereby increase in frequency. Thus, our data indicate that the benefits of these public goods include enhanced bacterial persistence and fitness during infection.

Staphylococcal dissemination into host tissue triggers a strong inflammatory response, attracting phagocytes to the site of infection to prevent microbial spread. Hence, for the indiscernible infectious foci to metamorphose into visible abscesses, *S. aureus* must avoid being killed by the infiltrating immune cells. To further characterize the benefit of coagulases, kidneys—representative of the pathology observed for the majority of the mice in each cohort—were evaluated via hematoxylin and eosin staining (Fig. 2).

**Fig. 2:**
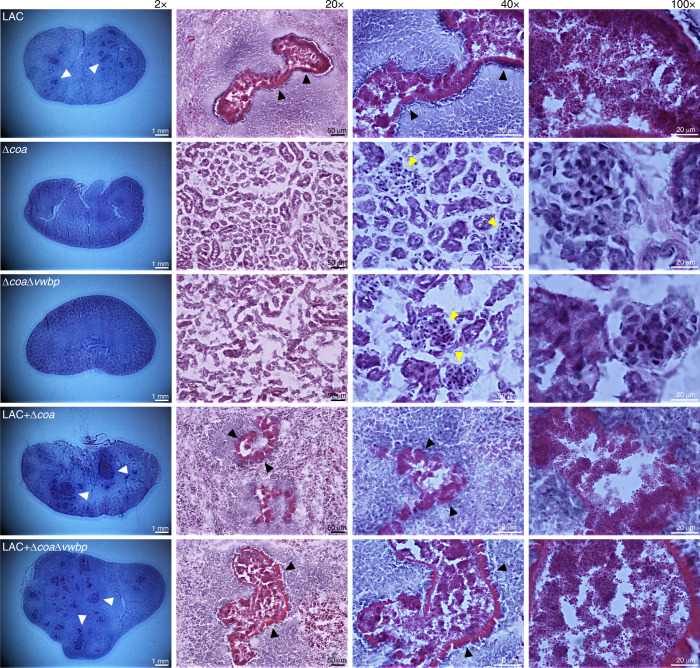
Coagulases generate protective shields around staphylococcal abscess communities. Kidneys were frozen in cryomatrix, thin-sectioned, stained with hematoxylin–eosin and images were captured by light microscopy and visualized with ×2, ×20, ×40 oil, and ×100 oil objectives. Renal tissues of mice infected with LAC, LAC & Δ*coa*, or LAC & Δ*coa*Δ*vwbp* had visible abscess lesions (×2 obj., white arrows). The staphylococcal abscess communities (×40 and ×100 obj.) were enclosed by an amorphous eosinophilic pseudocapsule (×20 and ×40 obj., black arrows), surrounded by a zone of dead/necrotic leukocytes (×20 obj.). The renal sections of mice infected with only cheats, Δ*coa* or Δ*coa*Δ*vwbp*, were absent of any characteristic abscess lesions (×2 obj.), displaying healthy tissue (×2 and ×20 obj.) and healthy leukocytes (×40 obj., yellow arrows, and ×100).

Mice monoinfected with LAC, and coinfected with LAC and Δ*coa*, or LAC and Δ*coa*Δ*vwbp* had abscess lesions in their kidneys (×2 obj.) (Fig. 2). These demarcated hypercellular regions had a central nidus of staphylococci (×100 obj.) enclosed within an amorphous pseudocapsule (×20 and ×40 obj.) and were circumscribed by zones of apparently dead immune cells. Conversely, this was not the case for the majority of the mice monoinfected with Δ*coa* or Δ*coa*Δ*vwbp*, as exemplified by the healthy renal tissue lacking abscess lesions (×2, ×20, and ×40 obj.) and the presence of intact immune cells (×40 and ×100 obj.) (Fig. 2). A simple explanation for this observation is that by triggering the ProT-mediated conversion of fibrinogen to fibrin, coagulases usurp the clotting cascade and form pseudocapsule barriers that hinder infiltrating immune cells from accessing the enclosed staphylococcal communities. Whereas in the absence of these public goods, cheats display defects in abscess formation (Figs. 1b and 2, and Supplementary Fig. S1a) and survival (Fig. 1e, f). However, when coinfected together with producers, cheats also become shielded (Fig. 2) and are therefore able to persist within the necropurulent foci and increase in frequency (Fig. 1e). Taken together, these results indicate that coagulases generate fibrin shields that protect both producers and cheats in a staphylococcal abscess community. In line with our previous in vitro and ex vivo observation^4^, this protection is most likely passively extended onto cheats when they co-aggregate together with producers, which places them near the public goods and ensures their survival throughout disease progression.

## Methods

### Bacterial strains and growth conditions

USA300 LAC and mutants in this background, Δ*coa* and Δ*coa*Δ*vwbp*, were used for this study, and have previously been described in detail^4^. Strains were grown in Tryptic soy broth (TSB) with 100 μg/mL rifampicin or streptomycin prior to infecting the mice. Enumeration of colony forming units (CFU) was done on selective Tryptic soy agar (TSA) infused with 20 μg/mL rifampicin or streptomycin.

### Retro-orbital injections in murine model

Strains were grown overnight at 37 °C in fresh TSB supplemented with rifampicin or streptomycin. Bacteria were centrifuged for 10 min at 6300 × *g*, washed, and resuspended in the same volume of sterile 1× PBS, repeated twice to rinse off all antibiotics. The optical densities of all overnight cultures were normalized. Injectates were prepared in 1× PBS as either monocultures or 1:1 ratio cocultures, vortexed for 3 min at ~2500 rpm, and placed at room temperature while preparing the mice for the procedure. Using the retro-orbital injection technique^27^, 6-week-old female Swiss Webster mice (Charles River Laboratories) were injected retro-orbitally with 100 μL of 1 × 10^6^ CFU/mL *S. aureus* suspensions in 1× PBS. On Day 15 post infection, mice were killed by sodium-pentobarbital injections and their kidneys and spleens were excised. The organs were gross-examined for signs of inflammation and surface abscess lesions and subsequently photographed at ×1.0 objective with a Stemi 2000-C stereomicroscope (Carl Zeiss) equipped with a DS-Fi1 camera (Nikon). Subsequently, the organs were weighed on an analytical balance, placed in 1 mL of 1× PBS inside tissue homogenizing tubes with 2 mm steel beads, homogenized by bead beating (FastPrep-24, MP Biomedical), vortexed, serially diluted, and 100 μL of homogenate was spread on selective TSA. Bacterial load was calculated as log_10_ CFU per gram of tissue.

### Hematoxylin and eosin staining of tissue sections

An independent set of approximately 15 kidneys and spleens from three animals in each cohort were placed in a Tissue-Tek vinyl specimen Cryomold (Sakura Finetek) containing a cryomatrix of OCT (optimum cutting temperature) compound (Thermo Fisher Scientific), and then immediately placed in a freezer at −80 °C to allow the OCT compound to solidify. Frozen sections were securely anchored using deep-waffled, large-face block holders (Electron Microscopy Sciences). Frozen OCT-embedded samples were sectioned using an OTF5000 cryostat (Bright Instrument Co., Ltd.) to a thickness of 12 μm for those imaged at ×2 objective, and 6 μm for those imaged at ×20 objective/N.A. 0.75, ×40 oil objective/N.A. 1.3, and ×100 oil objective/N.A. 1.3. Sections were directly transferred to Superfrost Plus microscope slides (Thermo Fisher Scientific) and stored at −20 °C until ready for visualization. Frozen sections were prepared for staining by air drying at room temperature for 5 min, then subjected to hematoxylin and eosin (H&E) staining using standard laboratory techniques and were mounted with Permount mounting medium (Fisher Scientific) before visualization. Mounted slides were then imaged by light microscopy with an Eclipse 80i microscope (Nikon), and images were captured with a DS-Fi1 camera (Nikon) equipped with a Digital Sight DS-U2 controller using NIS-Elements software version 3.00 SP7 (Nikon) and analyzed with the NIS-Elements Ar software version 4.50.00 (Nikon). Images at ×2 objective were captured using a Zeiss Stemi 2000-C stereomicroscope with a DS-Fi1 camera (Nikon).

### Ethics statement

All animal experiments involved a protocol (#16046) that was reviewed and approved by the Institutional Animal Care and Use Committee (IACUC). Animals are managed by the Texas Tech University Health Sciences Center (TTUHSC) Laboratory Animal Research Center (LARC), which is accredited by the Association for the Accreditation and Assessment of Laboratory Animal Care International, and National Institute of Health (NIH)-Office for Laboratory Animal Welfare. Animals are maintained per the applicable portions of the Animal Welfare Act and Regulations, and NIH-Animal Research Advisory Committee guidelines. Veterinary care is provided under the direction of the institutional veterinarians boarded by the American College of Laboratory Animal Medicine.

All procedures were performed by highly trained personnel. Animals were monitored for signs of morbidity and pain as specified by the IACUC: rapid respiration, slow, shallow or labored respiration; shock and ruffled fur; dehydration, inappetence and rapid weight loss; abnormal or hunched posture; animal not alert, abnormal movement; guarding reaction upon contact; vocalization when palpated or moved; self-mutilation, restlessness or lethargy. In addition, signs for judging infected animals to be moribund included any one of the following: complications associated with ocular pathology; impaired ambulation; evidence for muscle atrophy or emaciation. All animals were killed by sodium-pentobarbital injections, an approved method by the Panel on Euthanasia of the American Veterinary Medical Association.

### Statistical analyses

Data and statistical analyses were performed using R version 3.5.0^29^. Statistical tests used for the analysis of data are identified in the legend of each figure. Differences of *P* < 0.05 were considered significant.

### Reporting summary

Further information on research design is available in the Nature Research Reporting Summary linked to this article.

## Supplementary information

Supplementary Information

Reporting Summary

## Data Availability

Source data for figures will be made available upon request to the Section of Microbiology, University of Copenhagen upon publication.

## References

[1] Bartlett AH, Hulten KG (2010). Staphylococcus aureus pathogenesis: secretion systems, adhesins, and invasins. Pediatr. Infect. Dis. J..

[2] Lowy FD (1998). Staphylococcus aureus infections. N. Engl. J. Med..

[3] Bashore TM, Cabell C, Fowler V (2006). Update on infective endocarditis. Curr. Probl. Cardiol..

[4] Trivedi, U. et al. Staphylococcus aureus coagulases are exploitable yet stable public goods in clinically relevant conditions. *Proc. Natl .Acad. Sci. USA* 115,E11771–E11779 (2018). 10.1073/pnas.1804850115 PMC6294911 30463950

[5] Zajdel M, Wagrzynowicz Z, Jeljaszewicz J (1975). Action of staphylothrombin on bovine fibrinogen. Thromb. Res..

[6] Friedrich R (2003). Staphylocoagulase is a prototype for the mechanism of cofactor-induced zymogen activation. Nature.

[7] Bjerketorp J, Jacobsson K, Frykberg L (2004). The von Willebrand factor-binding protein (vWbp) of Staphylococcus aureus is a coagulase. FEMS Microbiol. Lett..

[8] Kroh HK, Panizzi P, Bock PE (2009). Von Willebrand factor-binding protein is a hysteretic conformational activator of prothrombin. Proc. Natl. Acad. Sci. USA.

[9] De NS (1950). Experimental pyelonephritis in the rabbit produced by staphylococcal infection. J. Pathol. Bacteriol..

[10] Freedman LR (1960). Experimental pyelonephritis. VI. Observations on susceptibility of the rabbit kidney to infection by a virulent strain of Staphylococcus aureus. Yale J. Biol. Med..

[11] Cheng AG, DeDent AC, Schneewind O, Missiakas D (2011). A play in four acts: Staphylococcus aureus abscess formation. Trends Microbiol..

[12] Gorbach SL (1995). Good and laudable pus. J. Clin. Invest..

[13] Tzianabos AO, Wang JY, Lee JC (2001). Structural rationale for the modulation of abscess formation by Staphylococcus aureus capsular polysaccharides. Proc. Natl. Acad. Sci. USA.

[14] Tzianabos AO, Kasper DL, Cisneros RL, Smith RS, Onderdonk AB (1995). Polysaccharide-mediated protection against abscess formation in experimental intra-abdominal sepsis. J. Clin. Invest..

[15] Wallenfang T, Bohl J, Kretzschmar K (1980). Evolution of brain abscess in cats formation of capsule and resolution of brain edema. Neurosurg. Rev..

[16] Russell DG (2008). Staphylococcus and the healing power of pus. Cell Host Microbe.

[17] Cheng AG (2009). Genetic requirements for Staphylococcus aureus abscess formation and persistence in host tissues. FASEB J..

[18] Cheng AG (2010). Contribution of coagulases towards Staphylococcus aureus disease and protective immunity. PLoS Pathog..

[19] Vandenesch F (1994). Coagulase deficiency in clinical isolates of Staphylococcus aureus involves both transcriptional and post-transcriptional defects. J. Med. Microbiol..

[20] Fonsale N (2004). Specific identification of Staphylococcus aureus by Staphychrom II, a rapid chromogenic staphylocoagulase test. J. Clin. Microbiol..

[21] Rotun SS (1999). Staphylococcus aureus with reduced susceptibility to vancomycin isolated from a patient with fatal bacteremia. Emerg. Infect. Dis..

[22] Wisniewska K, Garbacz K, Piechowicz L (2008). Genotypic screening of atypical Staphylococcus aureus strains isolated from clinical samples for presence of selected adhesin genes. Med. Mal. Infect..

[23] Młynarczyk G (1998). Coagulase-negative variants of methicillin-resistant Staphylococcus aureus subsp.aureus strains isolated from hospital specimens. Zentralblatt f.ür. Bakteriologie.

[24] Malinowski E, Lassa H, Klossowska A, Smulski S, Kaczmarowski M (2009). Atypical Staphylococcus aureus as an aetiological agent of mastitis in cows. B Vet. I Pulawy.

[25] Fox LK, Besser TE, Jackson SM (1996). Evaluation of a coagulase-negative variant of Staphylococcus aureus as a cause of intramammary infections in a herd of dairy cattle. J. Am. Vet. Med Assoc..

[26] Mancini M (2015). Head and neck veins of the mouse. a magnetic resonance, micro computed tomography and high frequency color doppler ultrasound study. PLoS ONE.

[27] Yardeni T, Eckhaus M, Morris HD, Huizing M, Hoogstraten-Miller S (2011). Retro-orbital injections in mice. Lab. Anim. (NY).

[28] Gresham HD (2000). Survival of Staphylococcus aureus Inside Neutrophils contributes to infection. J. Immunol..

[29] R Core Team. (2017). R: A language and environment for statistical computing.

