# Supplementary material for: Persistence and progression of staphylococcal infection in the presence of public goods
Source: NPJ Biofilms Microbiomes. 2020 Nov 27;6:55. doi: 10.1038/s41522-020-00168-2 (PMC7699630; doi:10.1038/s41522-020-00168-2)
Supplement: Supplementary file 1 — Supplementary Information [file 41522_2020_168_MOESM1_ESM.pdf]

Supplementary Information

Persistence and progression of staphylococcal infection in the presence of public goods

Urvish Trivedi[1], Cody Fell[2], Jonas S. Madsen[1], Jake Everett[2], Mette Burmølle[1], Kendra P. Rumbaugh[2], Søren J. Sørensen[1*]

[1] Section of Microbiology, Department of Biology, Faculty of Science, University of Copenhagen, 2100 Copenhagen, Denmark

[2] Department of Surgery, Texas Tech University Health Sciences Center, Lubbock, Texas 79430, United States of America

*Corresponding author: sjs@bio.ku.dk

Supplementary Figure 1.

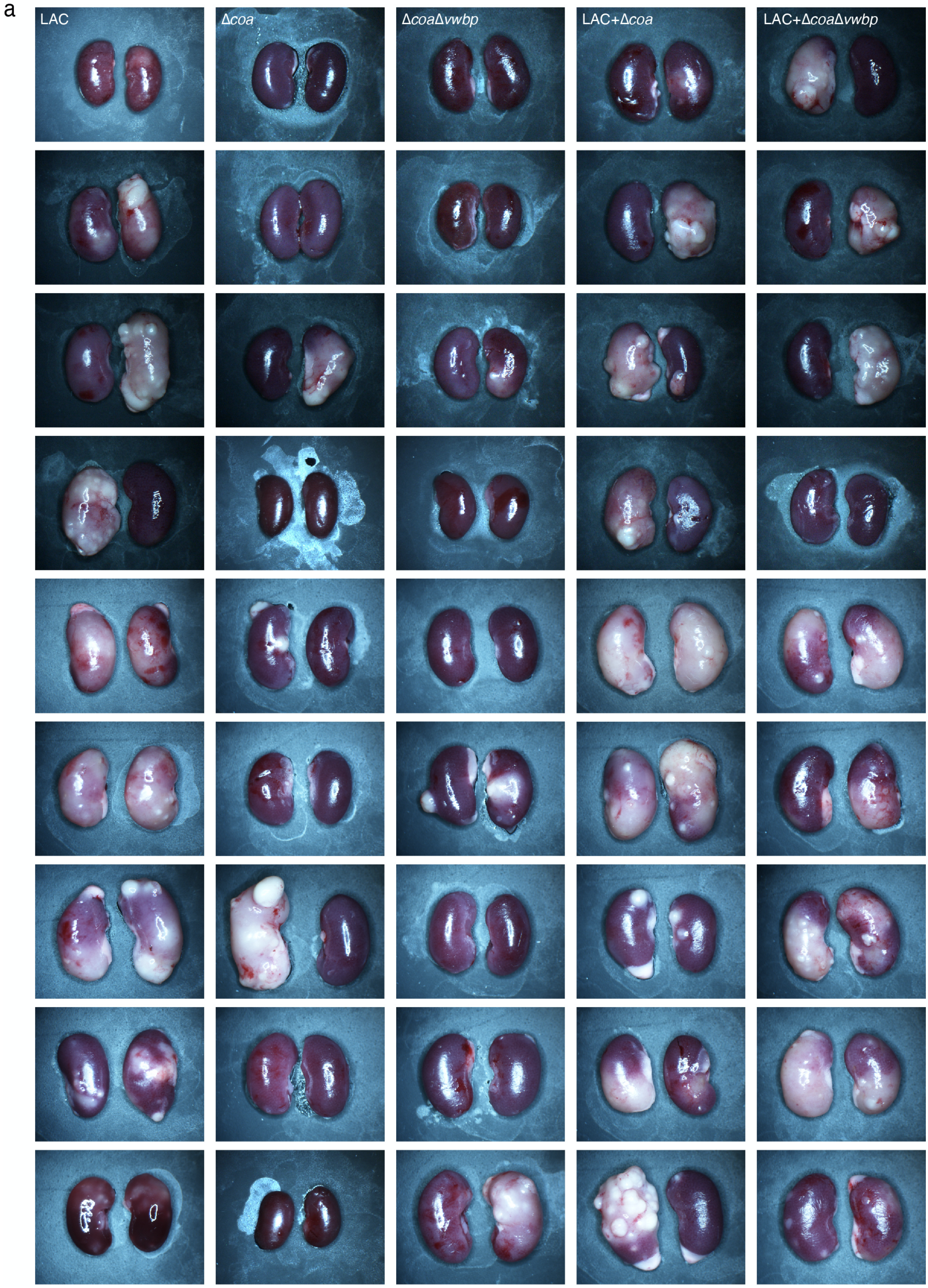

b

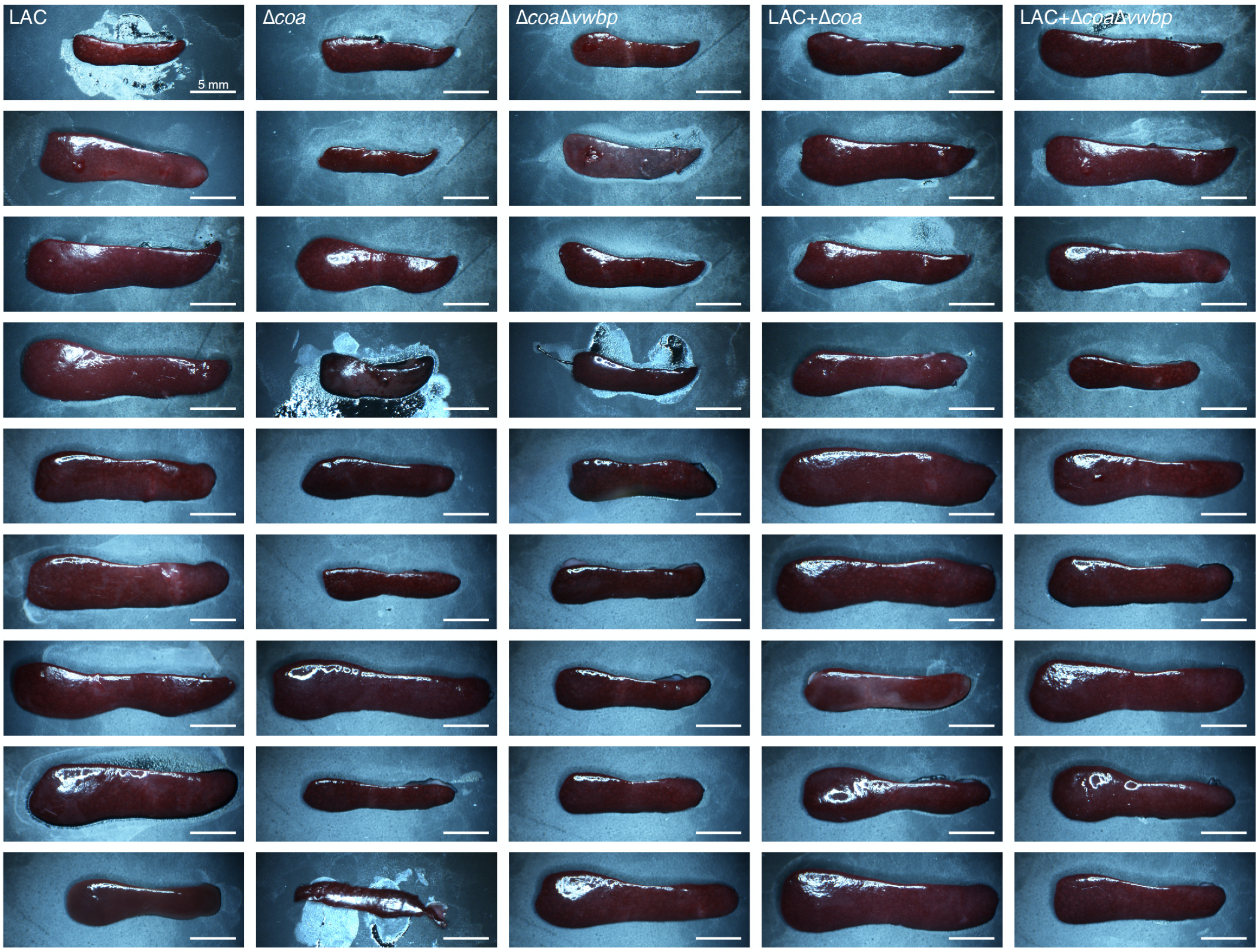

Supplementary Figure 1a and b. Contribution of coagulases towards renal abscesses and splenomegaly. Mice (n=9 for each cohort) were injected in their retro-orbital plexus with 1x10^{6} CFU of LAC, Δ*coa*, or Δ*coa*Δ*vwbp*; and cocultures of LAC and Δ*coa*, or LAC and Δ*coa*Δ*vwbp*. On Day 15 post-infection, the kidneys and spleens were excised, gross-examined, and photographed with a Stemi 2000-C stereomicroscope (Carl Zeiss) equipped with a DS-Fi1 camera (Nikon). (a) Image panels of all the kidneys harvested from infected animals corresponding to the data shown in Figure 1. The kidneys of animals infected with LAC, LAC and Δ*coa*, or LAC and Δ*coa*Δ*vwbp* have more abscesses and an overall worse pathology than those of animals infected with only Δ*coa*, or Δ*coa*Δ*vwbp*. (b) Image panels of all the spleens harvested from infected animals corresponding to the data shown in Figure 1. Spleens of animals infected with LAC, LAC and Δ*coa*, or LAC and Δ*coa*Δ*vwbp* were much larger than those for animals infected with only Δ*coa*, or Δ*coa*Δ*vwbp*. White scale bars = 5 mm.
