# Supplementary material for: Persistence and progression of staphylococcal infection in the presence of public goods
Source: NPJ Biofilms Microbiomes. 2020 Nov 27;6:55. doi: 10.1038/s41522-020-00168-2 (PMC7699630; doi:10.1038/s41522-020-00168-2)
Supplement: Supplementary file 2 — Reporting Summary [file 41522_2020_168_MOESM2_ESM.pdf]

nature research

Corresponding author(s): Søren J. Sørensen

Last updated by author(s): Feb 20, 2020

Reporting Summary

Nature Research wishes to improve the reproducibility of the work that we publish. This form provides structure for consistency and transparency in reporting. For further information on Nature Research policies, see Authors & Referees and the Editorial Policy Checklist.

Statistics

For all statistical analyses, confirm that the following items are present in the figure legend, table legend, main text, or Methods section.

n/a	Confirmed	
☐	☒	The exact sample size (n) for each experimental group/condition, given as a discrete number and unit of measurement
☐	☒	A statement on whether measurements were taken from distinct samples or whether the same sample was measured repeatedly
☐	☒	The statistical test(s) used AND whether they are one- or two-sided<br>*Only common tests should be described solely by name; describe more complex techniques in the Methods section.*
☒	☐	A description of all covariates tested
☒	☐	A description of any assumptions or corrections, such as tests of normality and adjustment for multiple comparisons
☐	☒	A full description of the statistical parameters including central tendency (e.g. means) or other basic estimates (e.g. regression coefficient) AND variation (e.g. standard deviation) or associated estimates of uncertainty (e.g. confidence intervals)
☐	☒	For null hypothesis testing, the test statistic (e.g. F, t, r) with confidence intervals, effect sizes, degrees of freedom and P value noted<br>*Give P values as exact values whenever suitable.*
☒	☐	For Bayesian analysis, information on the choice of priors and Markov chain Monte Carlo settings
☒	☐	For hierarchical and complex designs, identification of the appropriate level for tests and full reporting of outcomes
☐	☒	Estimates of effect sizes (e.g. Cohen's d, Pearson's r), indicating how they were calculated

Our web collection on statistics for biologists contains articles on many of the points above.

Software and code

Policy information about availability of computer code

Data collection	Images were captured using NIS-Elements software version 257 3.00 SP7 (Nikon).
Data analysis	Images were analysed using NIS-Elements Ar software version 4.50.00 (Nikon); data and statistical analyses were done using R 3.5.0.

For manuscripts utilizing custom algorithms or software that are central to the research but not yet described in published literature, software must be made available to editors/reviewers. We strongly encourage code deposition in a community repository (e.g. GitHub). See the Nature Research guidelines for submitting code & software for further information.

Data

Policy information about availability of data

All manuscripts must include a data availability statement. This statement should provide the following information, where applicable:

- Accession codes, unique identifiers, or web links for publicly available datasets
- A list of figures that have associated raw data
- A description of any restrictions on data availability

All data will be available upon request to the Section of Microbiology, UCPH after publication.

Field-specific reporting

Please select the one below that is the best fit for your research. If you are not sure, read the appropriate sections before making your selection.

☒ Life sciences ☐ Behavioural & social sciences ☐ Ecological, evolutionary & environmental sciences

For a reference copy of the document with all sections, see nature.com/documents/nr-reporting-summary-flat.pdf

Life sciences study design

All studies must disclose on these points even when the disclosure is negative.

Sample size	No statistical methods were used to predetermine sample size. Sample size was chosen based on previous studies and experience to the number that would be sufficient for analysis.
Data exclusions	No samples or data were excluded in this study.
Replication	For Figure 1, each experimental group consisted of 9 animals, where 4 to 5 animals were infected using independent inoculums; the data shown is the pooled data of the two independent inoculum trials. There was no statistical difference between the two inoculum trials (Wilcoxon, p=0.98).
Randomization	Mice were similar in age, close in weight, and were therefore randomly assigned to the experimental groups.
Blinding	Pathology measurements, analysis and scoring was single-blinded. Since animals needed to be housed in separate cages according to their group to avoid cross-contamination and needed to be properly labeled, blinding was not possible at the start of the study.

Reporting for specific materials, systems and methods

We require information from authors about some types of materials, experimental systems and methods used in many studies. Here, indicate whether each material, system or method listed is relevant to your study. If you are not sure if a list item applies to your research, read the appropriate section before selecting a response.

Materials & experimental systems

n/a	Involved in the study	
☒	☐	Antibodies
☒	☐	Eukaryotic cell lines
☒	☐	Palaeontology
☐	☒	Animals and other organisms
☒	☐	Human research participants
☒	☐	Clinical data

Methods

n/a	Involved in the study	
☒	☐	ChIP-seq
☒	☐	Flow cytometry
☒	☐	MRI-based neuroimaging

Animals and other organisms

Policy information about studies involving animals; ARRIVE guidelines recommended for reporting animal research

Laboratory animals	Six-week old female Swiss Webster mice (Charles River Laboratories) were used in this study.
Wild animals	This study did not involve wild animals.
Field-collected samples	This study did not involve field-collected samples.
Ethics oversight	All animal studies were performed in accordance with protocols reviewed and approved by the Institutional Animal Care and Use Committee (IACUC) at the Texas Tech University Health Sciences Center (TTUHSC) Laboratory Animal Research Center (LARC).

Note that full information on the approval of the study protocol must also be provided in the manuscript.
